# To the Editors of the Medical and Physical Journal

**Published:** 1805-06-01

**Authors:** Charles Platt

**Affiliations:** Great Surry Street


### To the Editors of the Medical and Physical Journal.

Gentlemen,

"Sic ego, majoris fugiens opprobria culpæ

"Frontis ad urbanæ descendi præmia."

FEW possess less inclination to enter into a polemical controversy on the construction of an obscure passage, than myself; but as I am accused of "being guilty of misrepresenting the meaning of an author,"* I solicit your indulgence to an explanation.

Mr. Whately, in a publication replete with much ingenuity, has endeavoured to inculcate the doctrine that gonorrhœa venerea is frequently attended with ulcerations in the urethra. In confirmation of this opinion, he has not only appealed to his own experience, in which he asserts he has had occular demonstration of their existence, but he further corroborates this opinion, by reference to some respectable authorities.

I will not question the accuracy of his observations, nor can I doubt the respectability of his authorities. That ulcers occasionally present themselves in the urethra, will not be denied; but it does not follow that these ulcerations are the necessary consequence of a gonorrhœa. The ingenious gentleman and myself, differ not so much about the *existence* of ulcers, as we do about their *production*. I have elsewhere,† briefly enumerated the various causes that may be supposed to contribute to this diseased state of the urethra; but it is extremely dubious, whether gonorrhœa may be considered as one.

At the present improved period of our anatomical knowledge, it will scarcely be doubted but that a puriform matter may be secreted from an inflamed surface, without any disorganization of structure. In catarrhal affections attended with an increased discharge from the Schniederian membrane, few, I believe, suspect the existence of ulceration in that delicate membrane: so, in the gonorrhœa, the dissection of executed criminals, as well as a variety of other cases, clearly demonstrate, after the most accurate investigation, that ulcerations in the urethra

---

* Vide Medical and Physical Journal, No. 74, p. 323.

† Platt's Inquiry into the Efficacy of Oxygen in the Cure of Syphilis, p. 20.

thra during this stage of the disease, rarely ever occurs.

If more correct information than I have hitherto acquired, should detect the fallacy of my present opinion, I will willingly relinquish it; till then, a steady adherence to it, will not, I trust, be considered too pertinacious.

I am, &c.

*Great Surry Street, May* 1, 1805. CHARLES PLATT.

---

